# Nuclear magnetic resonance spectroscopy data of isolated compounds from *Acacia farnesiana* (L) Willd fruits and two esterified derivatives

**DOI:** 10.1016/j.dib.2018.12.008

**Published:** 2018-12-07

**Authors:** Erika Hernández-García, Abraham García, Francisco G. Avalos-Alanís, Verónica M. Rivas-Galindo, Claudia Delgadillo-Puga, María del Rayo Camacho-Corona

**Affiliations:** a Universidad Autónoma de Nuevo León, Facultad de Ciencias Químicas, Av. Universidad S/N, Ciudad Universitaria, CP 66451 San Nicolás de los Garza, Nuevo León, Mexico; b Universidad Autónoma de Nuevo León, Facultad de Medicina, Av. Madero S/N, Col. Mitras Centro, CP 64460 Monterrey, Nuevo León, Mexico; c Instituto Nacional de Ciencias Médicas y Nutrición Salvador Zubirán, Av. Vasco de Quiroga No. 15, Col. Belisario Domínguez Sección XVI, CP 14080 Ciudad de México, Mexico

## Abstract

In the present article we describe the spectroscopic data of ^1^H and ^13^C Nuclear Magnetic Resonance of 11 compounds including: Nine natural products from the hexanic-chloroformic and methanolic extracts of *Acacia farnesiana* fruit and two esterified derivatives (22E-stimasta-5,22-dien- 3β-acetyl and methyl 3,4,5-triacetyloxybenzoate). Data linked to the research work entitled "Chemical composition of fruits of *Acacia farnesiana* (L) Willd and its activity against *Mycobacterium tuberculosis* and dysentery bacteria" (Hernández et al., 2019) [1].

**Specifications table**

**Table:** 

Subject area	*Phytochemistry*
Type of data	*NMR spectra figures*
How data was acquired	*NMR equipment Bruker* AVANCE III HD *400 MHz*
Data format	*Analysed*
Experimental factors	*Dissolution of the compounds in deuterated solvent CDCl*_*3*_*, DMSO-d*_*6*_*, Acetone-d*_*6*_*and D*_*2*_*O*
Experimental features	*NMR*^1^*H and*^13^*C chemical shift, integration, coupling constants and multiplicity*
Data source location	*Facultad de Ciencias Químicas*
	*Universidad Autónoma de Nuevo León. Guerreo y Progreso S/N. Col. Treviño, Monterrey, Nuevo León, México. C.P. 64570.*
Data accessibility	*All data are available in this document.*
Related research article	Hernández, E., Garza, E., García, A., Avalos, F.G., Rivas, V. M., Rodríguez, J., Alcántar, V. M., Delgadillo, C., Camacho M. R. Chemical composition of *Acacia farnesiana* (L) wild fruits and its activity against *Mycobacterium tuberculosis* and dysentery bacteria. *J. Ethnopharmacol* 2019 230: 74–80 [1].

**Value of the data**

• The spectroscopic characterization of natural products reported in this article is important in the metabolic chemical characterization processes of plants of the same family, genus or different plant species.
• It is possible the chracterization of new o related phytochemicals by comparision with the provided spectroscopic data.

## 1 Data

^1^H and ^13^C Nuclear Magnetic Resonance techniques allowed the characterization of isolated compounds from the hexanic, chloroformic and methanolic extracts of *Acacia farnesiana* and esterified derivatives. NMR spectra data is shown, as well as the detailed description of the spectroscopic signals (chemical shift, integration, coupling constants, multiplicity and signal assignment), see Fig. 1, Fig. 2, Fig. 3, Fig. 4, Fig. 5, Fig. 6, Fig. 7, Fig. 8, Fig. 9, Fig. 10, Fig. 11, Fig. 12, Fig. 13, Fig. 14, Fig. 15, Fig. 16, Fig. 17, Fig. 18, Fig. 19, Fig. 20, Fig. 21, Fig. 22 with this article.

**Fig. 1:**
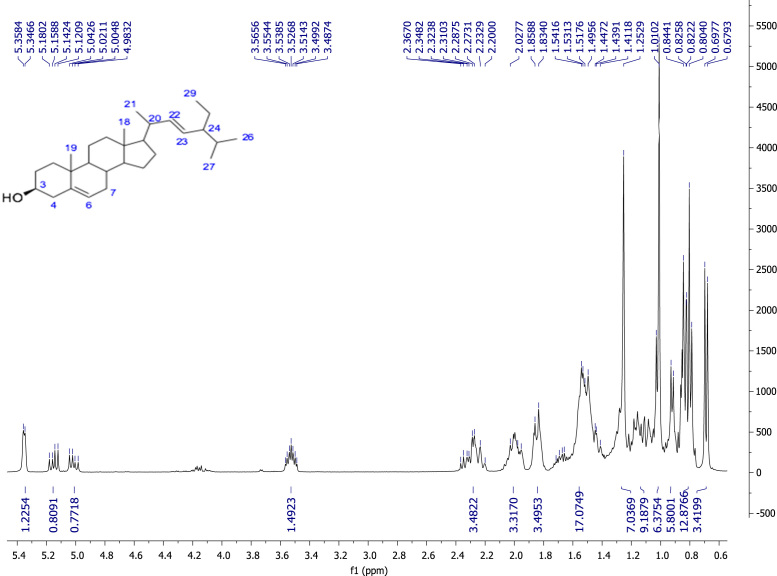
22E-stimasta-5,22-dien-3β-ol, NMR ^1^H (400 MHz CDCl_3_) δ ppm: 0.69 (s, 3H, Me-18), 0.79 (d, *J*=6.92 Hz, 3H, Me-27), 0.80 (t, *J*=7.1 Hz, 3H, Me-29), 0.83 (d, *J*=7.32 Hz, 3H, Me-26), 0.86 (d, *J*=3.8 Hz, 2H, H-28), 0.92 (d, *J*=6.4 Hz, 2H, H-9, H-24), 1.01 (s, 3H, Me-19), 1.02 (d, *J*=7. 72 Hz, 3H, Me-21), 1.10 (m, 1H, H-14), 1.04 (m, 2H, H-1), 1.07 (m, 2H, H-15), 1.11 (m, 1H, H-14), 1.13 (m, 1H, H-17), 1.16 (m, 1H, H-12), 1.28 (m, 1H, H-16), 1.41 (m, 1H, H-20), 1.53 (m, 2H, H-7), 1.54 (m, 1H, H-11), 1.83 (m, 1H, H-25), 1.84 (m, 2H, H-2), 1.85 (m, 1H, H-16), 1.99 (m, 1H, H-8), 2.0 (m, 2H, H-12), 2.28 (m, 2H, H-4), 3.52 (m, 1H, H-3), 5.01 (dd, *J*=15.1, 8.6 Hz, 1H, H-23), 5.15 (dd, *J*=15.1, 8.5 Hz, 1H, H-22), 5.35 (brd, *J*=4.72 Hz, 1H, H-6).

**Fig. 2:**
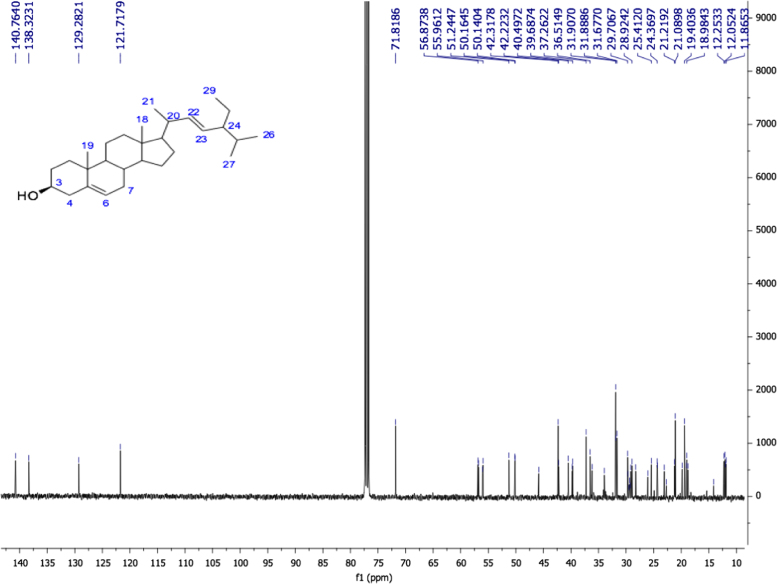
22E-stimasta-5,22-dien-3β-ol, NMR ^13^C (100 MHz, CDCl_3_) δ ppm: 12.05 (C18), 12.25 (C29), 19.03 (C27), 19.40 (C19), 21.08 (C11, C26), 21.21 (C21), 24.36 (C15), 25.41 (C28), 28.92 (C16), 31.67 (C2), 31.88 (C7, C8), 31.90 (C25), 36.51 (C10), 37.26 (C1), 39.78 (C12), 40.49 (C20), 42.22 (C13), 42.31 (C4), 50.14 (C9), 51.24 (C24), 55.96 (C17), 56.87 (C14), 71.81 (C3), 121.71 (C6), 129.28 (C23), 138.32 (C22), 140.76 (C5).

**Fig. 3:**
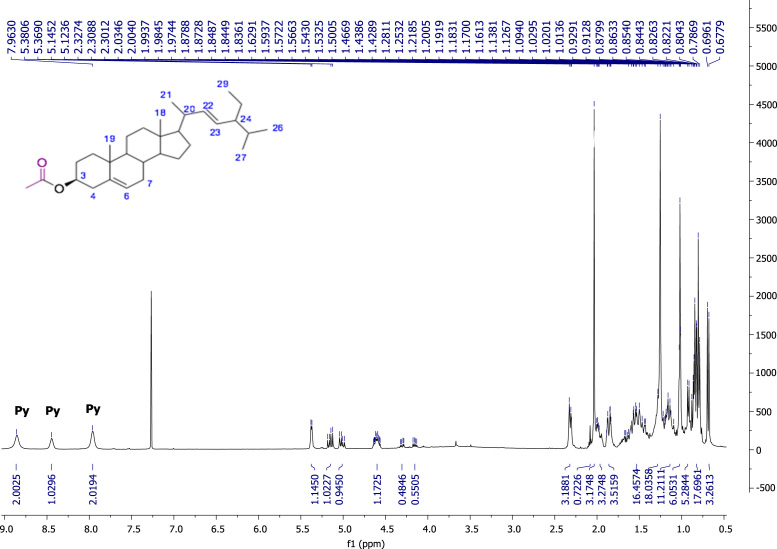
22E-stimasta-5,22-dien-3β-acetyl, NMR ^1^H (400 MHz, CDCl_3_) δ ppm: 0.69 (s, 3H, Me-18), 0.79 (d, *J*=6.96 Hz, 3H, Me-27) 0.80 (t, *J*=7.04 Hz, 3H, Me-29), 0.82 (d, *J*=1.74 Hz, 2H, H-28), 0.83 (d, *J*=7.2 Hz, 3H, Me-26),), 0.91 (m, 1H, H-24) 0.92 (d, *J*=6.54 Hz, 1H, H-9), 1.02 (s, 3H, Me-19), 1.021 (d, *J*=6.36 Hz, 3H, Me-21), 1.12 (m, 1H, H-14), 1.13 (m, 2H, H-15), 1.16 (m, 2H, H-1), 1.17 (m, 1H, H-17), 1.18 (m, 1H, H-12), 1.28 (m, 1H, H-16), 1.42 (m, 1H, H-20), 1.53 (m, 2H, H-11), 1.54 (m, 2H, H-7), 1.83 (m, 1H, H-25), 1.84 (m, 2H, H-2), 1.87 (m, 1H, H-16), 1.98 (m, 1H, H-8), 1.99 (m, 2H, H-12), 2.03 (s, 3H, CH_3_CO), 2.32 (m, 2H, H-4), 4.6 (m, 1H, H-3), 5.01 (dd, *J*=15.16, 8.64 Hz, 1H, H-23), 5.15 (dd, *J*=15.16, 8.6 Hz, 1H, H-22), 5.37 (brd, *J*=4.64, 1H, H-6).

**Fig. 4:**
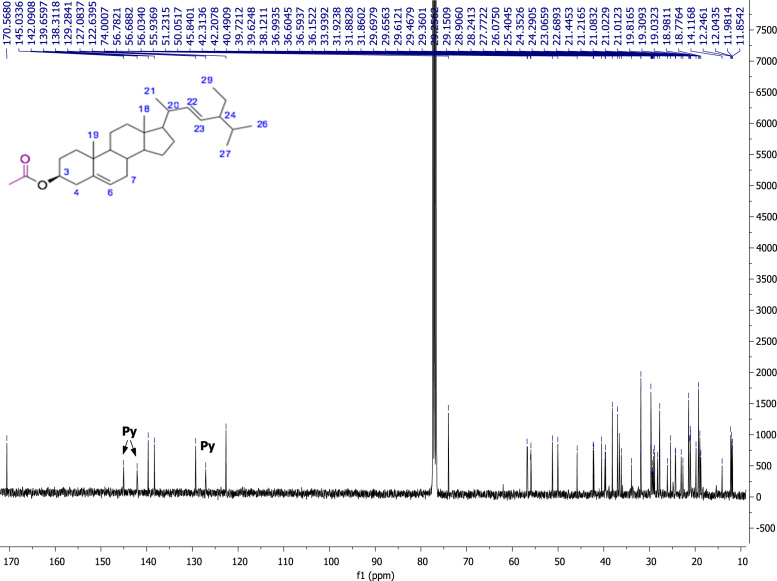
22E-stimasta-5,22-dien-3β-acetyl. NMR ^13^C (100 MHz, CDCl_3_) δ ppm: 12.04 (C18), 12.24(C29), 18.98 (C27), 19.30 (C19), 21.01 (C11), 21.08 (C26), 21.21 (C21), 21.44 (CH_3_CO), 24.35 (C15), 25.40 (C28), 27.77 (C2), 28.90 (C16), 31.86 (C7, C8), 31.88 (C25), 36.59 (C10), 36.99 (C1), 38.12 (C4), 39.62 (C12), 40.49 (C20), 42.20 (C13), 50.05 (C9), 51.23 (C24), 55.93 (C17), 56.78 (C14), 74.0 (C3), 122.63 (C6), 129.28 (C23), 138.31 (C22), 139.65 (C5), 170.56 (CH_3_CO).

**Fig. 5:**
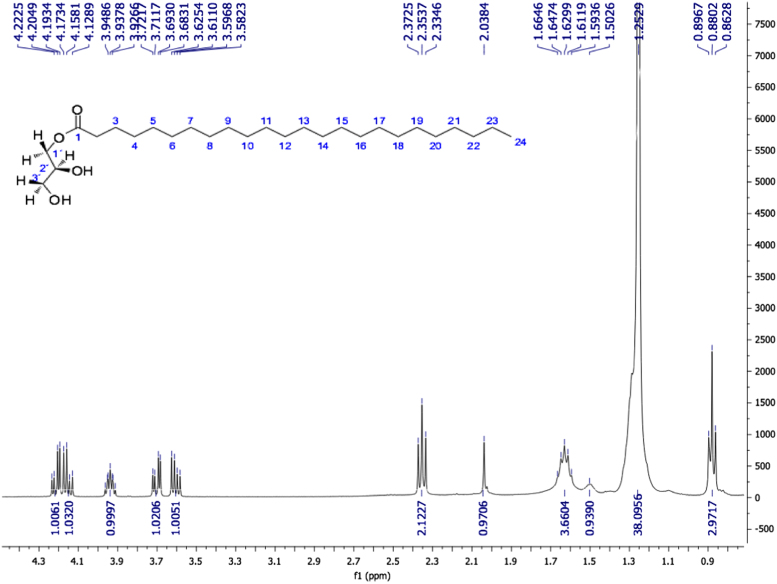
Tetracosanoic acid (2S)-2, 3-dihydroxypropyl ester, NMR ^1^H (400 MHz, CDCl_3_) δ ppm: 0.88 (t, J=6.78 Hz, 3H, Me-24), 1.25 (sa, 38H, (CH_2_)_19_, C4-C22), 1.50 (sa, 1H, OH-3´), 1.63 (m, 4H, H-3, H-23), 2.04 (s, 1H, OH-2´), 2.35 (t, *J*=7.58 Hz, 2H, H-2), 3.60 (dd, *J*=11.46, 5.78 Hz, 1H, H-3´β), 3.70 (dd, *J*=11.46, 3.98 Hz, 1H, H-3´α), 3.94 (m, 1H, H-2´), 4.15 (dd, *J*=11.68, 6.12 Hz, 1H, H-1´β), 4.21 (dd, *J*=11.64, 4.6 Hz, 1H, H-1´α).

**Fig. 6:**
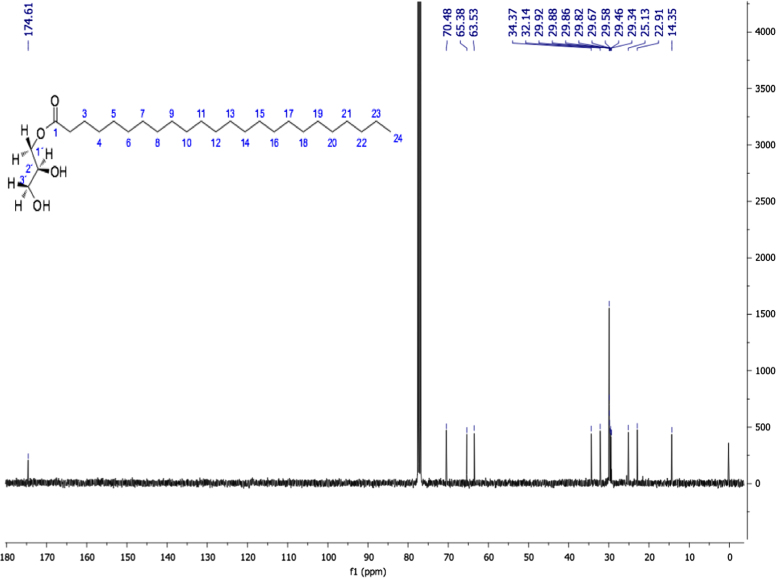
Tetracosanoic acid (2*S*)-2, 3-dihydroxypropyl ester, NMR ^13^C (100 MHz, CDCl_3_) δ ppm: 14.35 (C24), 22.91 (C23), 25.13 (C3), 29.34 (C4), 29.46 (C5), 29.58 (C8), 29.67 (C9), 29.82 (C21), 29.86 (C6), 29.88 (C7), 29.92 (C10-C20), 32.14 (C22), 34.37 (C2), 63.53 (C3´), 65.38 (C1´), 70.48 (C2´), 174.61 (C1).

**Fig. 7:**
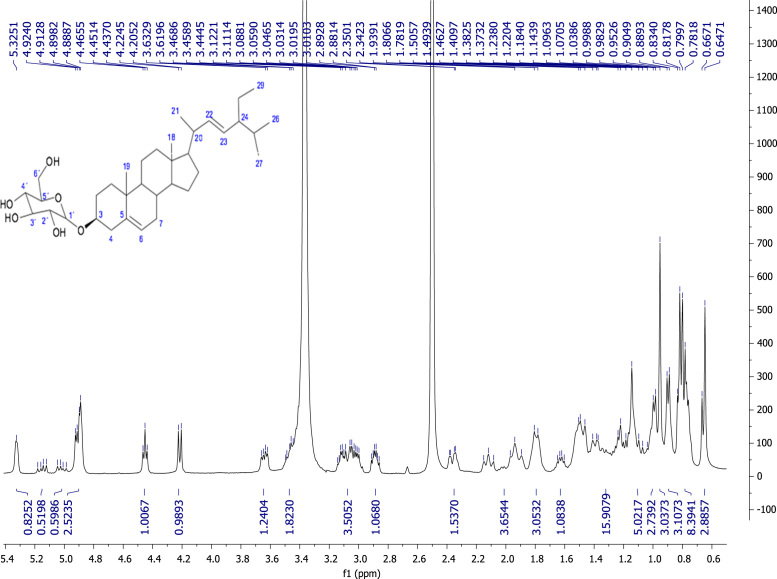
Stigmasta-5,22-dien-3β-O-D-glucopyranoside, NMR ^1^H (400 MHz, DMSO-d_6_) δ ppm: 0.64 (s, 3H, Me-18), 0.79 (t, *J=* 7.40 Hz, 3H, Me-29), 0.80 (d, *J=* 7.64 Hz, 3H, Me-27), 0.81 (m, 1H, H-9), 0.83 (m, 1H, H-24), 0.89 (d, *J=* 6.24 Hz, 3H, Me-26), 0.95 (s, 3H, Me-19), 0.99 (d, *J=* 6.36 Hz, 3H, Me-21), 1.03 (m, 1H, H-17), 1.07 (m, 2H, H-15), 1.09 (m, 1H, H-9), 1.14 (m, 2H, H-12), 1.19 (d, *J*= 7.1 Hz, 1H, H-4), 1.22 (m, 2H, H-11), 1.37 (m, 2H, H-2), 1.40 (m, 1H, H-20), 1.46 (m, 1H, H-25), 1.49 (m, 2H, H-7), 1.62 (dd, *J=* 6.4, 11.6, 1H, H-8), 1.78 (m, 1H, H-16), 1.80 (m, 1H, H-4), 1.93 (m, 1H, H-16), 2.11 (m, 1H, H-1), 2.36 (dd, *J=* 3.0, 13.3 Hz, 1H, H-1), 2.88 (m, 1H, H-2´), 3.01 (m, 2H, H-5´), 3.04 (m, 2H, 4´), 3.11 (m, 1H, H-3´), 3.46 (m, 1H, H-3), 3.48 (m, 1H, H-6´a), 3.63 (dd, *J=* 10.7, 5.4 Hz, 1H, H-6´b), 4.21 (d, *J=* 7.72 Hz, 1H, H-1´), 4.45 (t, *J=* 5.6 Hz, 1H, OH-6´), 4.88 (sa, 1H, OH-4´), 4.89 (sa, 1H, OH-2´), 4.91 (d, *J* = 4.5 Hz, 1H, OH-3´), 5.01 (dd, *J=* 15, 8.72, Hz, 1H, H-23), 5.15 (dd, *J=* 15.04, 8.62 Hz, 1H, H-22), 5.32 (sa, 1H, H-6).

**Fig. 8:**
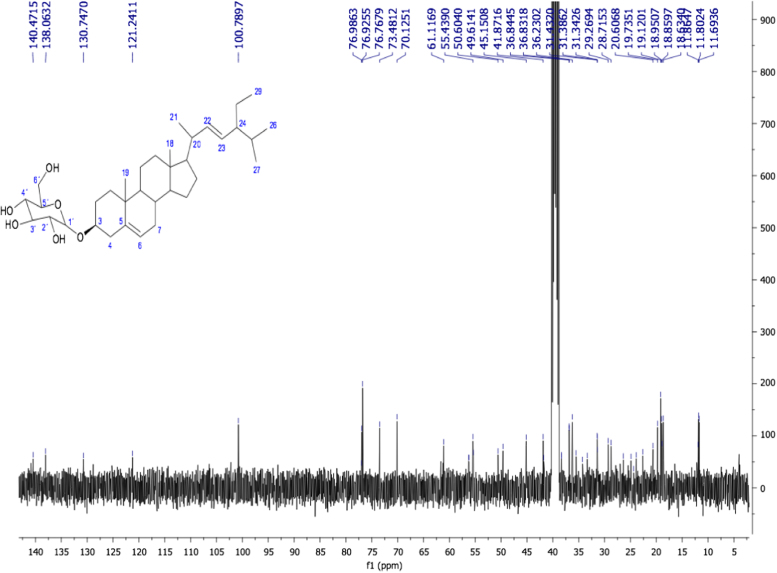
Stigmasta-5,22-dien-3β-O-D-glucopyranoside. NMR ^13^C (100 MHz, DMSO-d_6_): δ (ppm): 11.69 (C29), 11.80 (C18), 18.63 (C21), 18.85 (C27), 18.95 (C19), 19.12 (C26), 22.62 (C11), 23.88 (C28), 24.88 (C15), 29.26 (C16), 31.38 (C7, C8), 31.43 (C24, C25), 33.35 (C2), 35.49 (C20), 36.23 (C10), 36.83 (C4), 38.30 (C1), 39 (C12), 41.87 (C13), 49.61 (C9), 55.43 (C17), 56.27 (C14), 61.11 (C6´), 70.12 (C2´), 73.48 (C4´), 76.76 (C5´), 76.92 (C3´), 76.98 (C3), 100.78 (C1´), 121.24 (C6), 130.74 (C23), 138.06 (C22), 140.47 (C5).

**Fig. 9:**
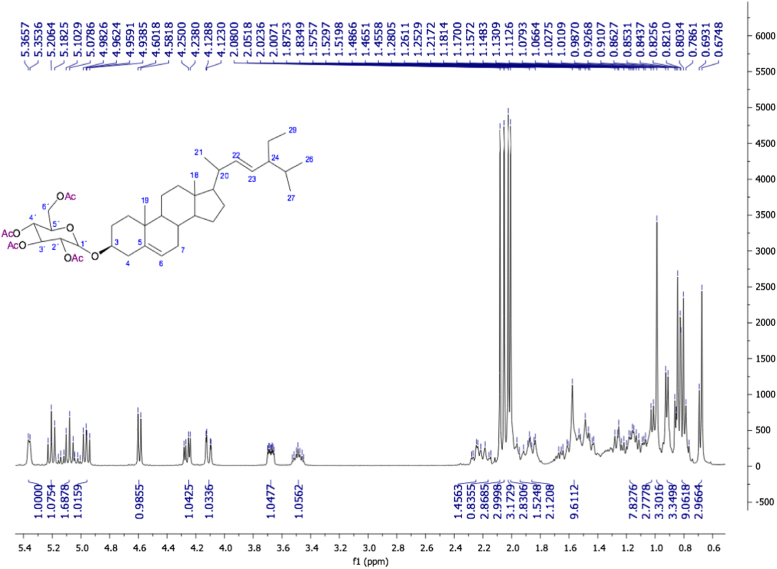
Stigmasta-5,22-dien-3β-O-D-tetraacetylglucopyranoside, NMR ^1^H (400 MHz, CDCl_3_) δ ppm: 0.67 (s, 3H, Me-18), 0.80 (t, *J=*7.24 Hz, 3H, Me-29), 0.83 (d, *J=*7.04 Hz, 3H, Me-27), 0.91 (d, *J=*6.44 Hz, 3H, Me-26), 0.98 (s, 3H, Me-19), 1.02 (d, *J=*6.64 Hz, 3H, Me-21), 2.00 (s, 3H, CH_3_CO-3´), 2.02 (s, 3H, CH_3_CO-2´), 2.05 (s, 3H, CH_3_CO-4´), 2.08 (s, 3H, CH_3_CO-6´), 3.48 (m, 1H, H-3), 3.67 (m, 1H, H-2´), 4.1 (dd, *J=*12.2, 2.88 Hz, 1H, H-6´a), 4.26 (dd, *J=*12.22, 4.82 Hz, 1H, H-6´b), 4.59 (d, *J=*8.0 Hz, 1H, H-1´), 4.96 (t, *J=* 9.48 Hz, 1H, H-3´), 5.03 (dd, *J=* 14.16, 5.56 Hz, 1H, H-23), 5.07 (t, *J=* 9.68 Hz, 1H, H-5´), 5.13 (dd, *J=*15.16, 6.52 Hz, 1H, H-22), 5.20 (t, *J=*9.52 Hz, 1H, H-4´) 5.36 (da, *J=*4.84 Hz, 1H, H-6).

**Fig. 10:**
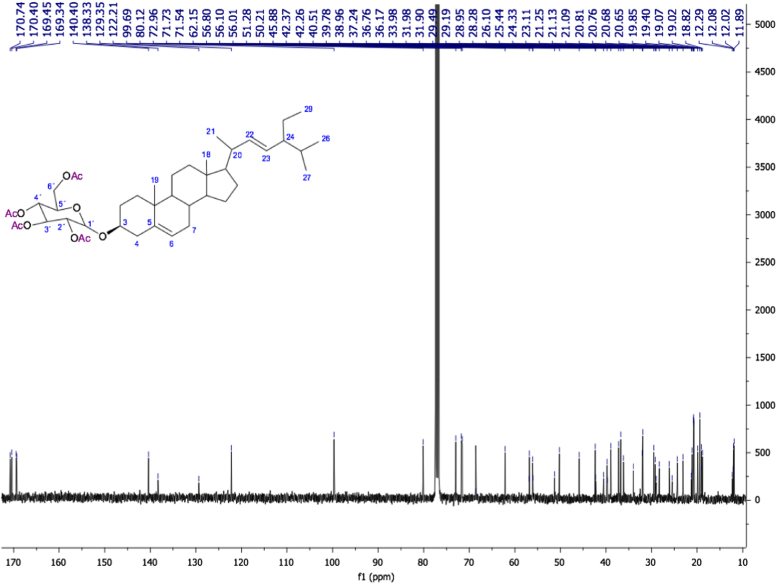
Stigmasta-5,22-dien-3β-O-D-tetraacetylglucopyranoside. NMR ^13^C (100 MHz, CDCl_3_) δ ppm: 11.89(C29), 12.02 (C18),18.81 (C21),19.07(C27), 19.39 (C19), 19.85 (C26), 20.65 (CH_3_CO-6´), 20.68 (CH_3_CO-4´), 20.76 (CH_3_CO-3´), 20.80 (CH_3_CO-2´), 21.08 (C11), 23.10 (C28), 24.33 (C15), 28.27 (C16), 29.48 (C24), 31.90 (C8, C25), 31.98 (C7), 33.98 (C-2), 36.16 (C20), 36.76 (C10), 37.23 (C1), 38.95 (C4), 39.77 (C12), 42.36 ( C13), 50.20 (C9), 56.09 (C17), 56.79 (C14), 62.15 (C6´), 68.53 (C4´), 71.54 (C3´), 71.73 (C5´), 72.96 (C3), 80.12 (C2´), 99.68 (C1´), 122.20 (C6), 129.34 (C23), 138.32 (C22), 140.40 (C5), 169.34 (CH_3_CO-3´), 169.44 (CH_3_CO-4´), 170.40 (CH_3_CO-2´), 170.74 (CH_3_CO-6´).

**Fig. 11:**
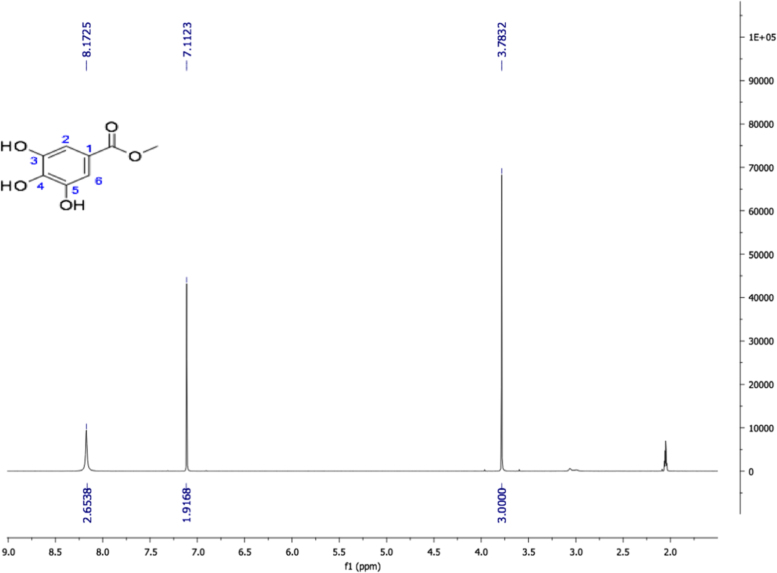
Methyl gallate, NMR ^1^H (400 MHz, Acetone-d_6_) δ ppm: 3.78 (s, 3H, OMe), 7.11 (s, 2H, H-2, H-6), 8.17 (s, 3H, OH).

**Fig. 12:**
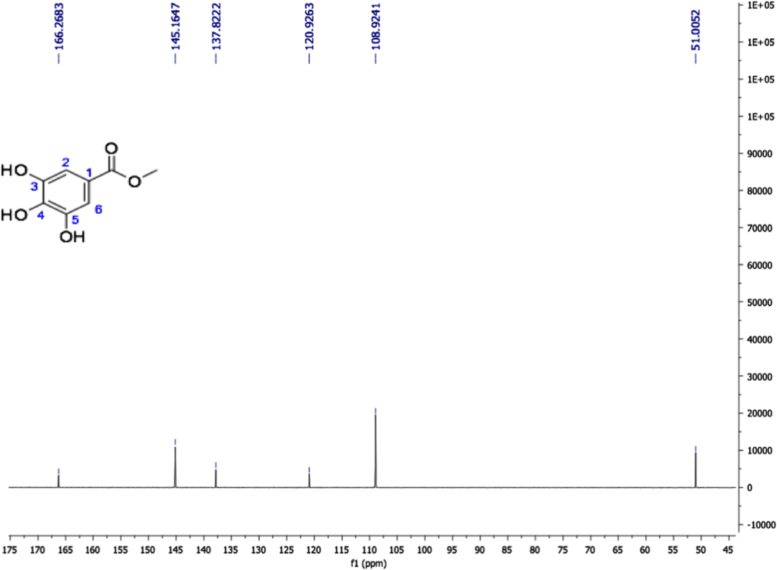
Methyl gallate, NMR ^13^C (100 MHz, Acetone-d_6_) δ ppm: 51.01 (OMe), 108.92 (C2, C6), 120.93 (C1), 137.82 (C4), 145.16 (C3, C5), 166.27 (COOR).

**Fig. 13:**
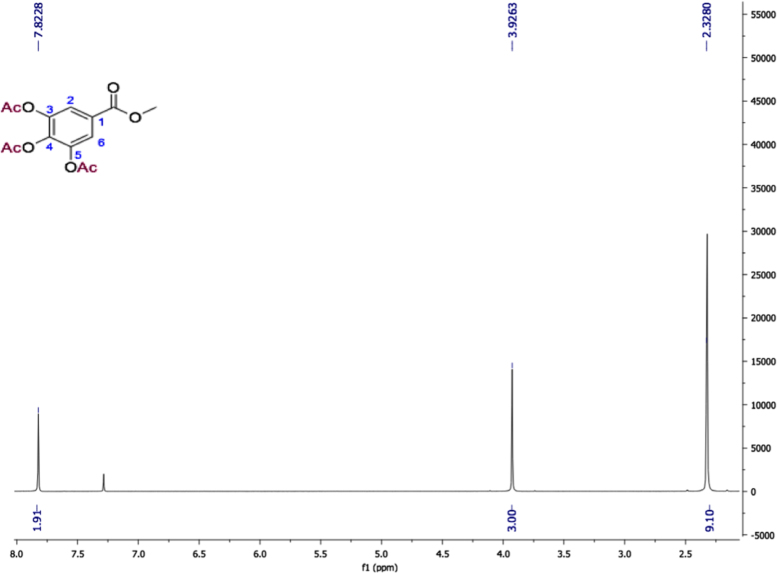
Methyl 3,4,5-triacetyloxybenzoate, NMR ^1^H (400 MHz, CDCl_3_) δ ppm: 2.32 (s, 9H, 3x CH_3_CO), 3.92 (s, 3H, OMe), 7.82 (s, H-2, H 6).

**Fig. 14:**
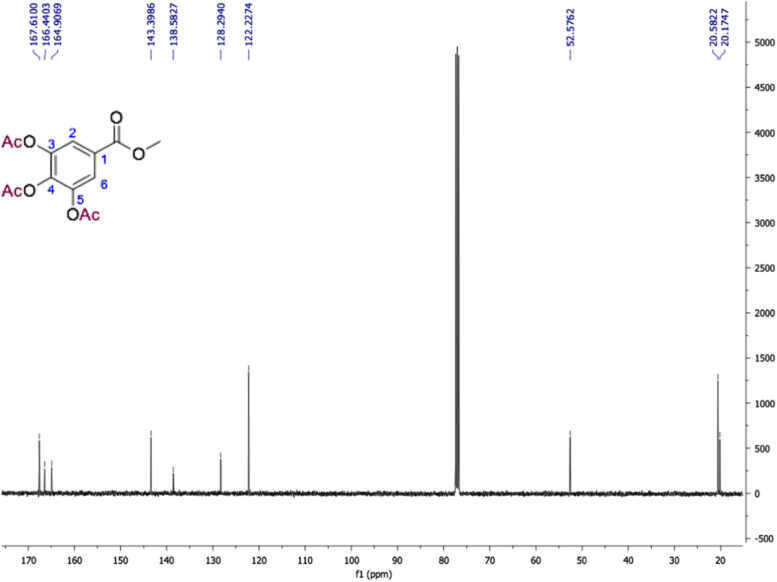
Methyl 3,4,5-triacetyloxybenzoate, NMR ^13^C (100 MHz, CDCl_3_) δ ppm: 20.17 (CH_3_CO-4), 20.58 (CH_3_CO-3, CH_3_CO-5), 52.57 (OCH_3_), 122.22 (C2, C6), 128.29 (C1), 138.58 (C4), 143.39 (C3, C5), 164.90 (CH_3_CO-1), 166.44 (CH_3_CO-4), 167.61 (CH_3_CO-3, CH_3_CO-5).

**Fig. 15:**
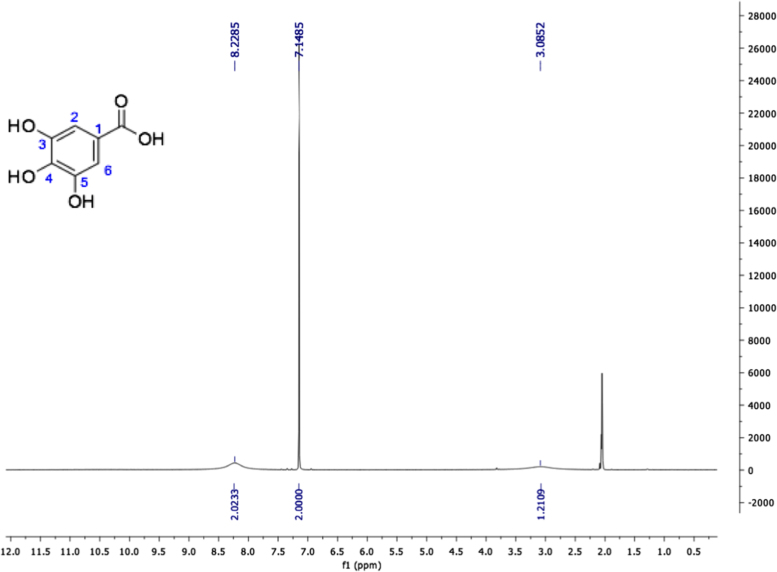
Gallic acid, NMR ^1^H (400 MHz, Acetone-d_6_) δ ppm: 3.08 (sa, 4H, OH-4), 7.14 (s, 2H, H-2, H-6), 8.22 (sa, 2H, OH-3, OH-5).

**Fig. 16:**
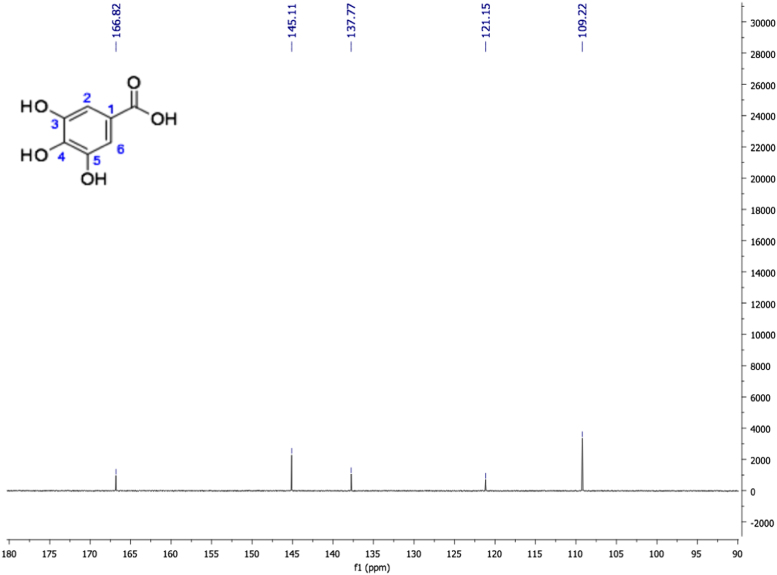
Gallic acid, NMR ^13^C (100 MHz, Acetone-d_6_) δ ppm: 109.22 (C2, C6), 121.15 (C1), 137.77 (C4), 145.11 (C3, C5), 166.82 (COOH).

**Fig. 17:**
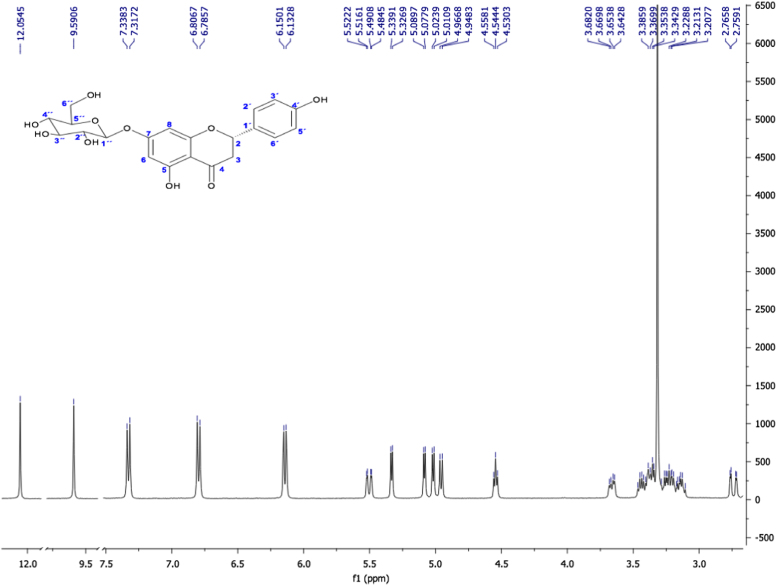
(2S) -Naringenin 7-O-β-D-glucopyranoside, NMR ^1^H (400 MHz, DMSO-d_6_) δ ppm: 2.73 (dd, *J=* 17.1, 2.62 Hz, 1H, H-3β), 3.14 (m, 1H, H-3α), 3.22 (m, 2H, H-4´´, H-2´´), 3.37 (m, 2H, H-3´´, H-5´´), 3.42 (dd, *J=* 11.68, 5.64 Hz, 1H, H-6a´´), 3.65 (dd, *J=* 11.04, 4.68 Hz, 1H, H-6b´´), 4.54 (t, *J=* 5.56, 1H, OH-6”), 4.95 (d, *J=* 7.4 Hz, 1H, H-1´´), 5.01 (d, *J=* 5.2 Hz, 1H, OH-4´´), 5.08 (d, *J=* 4.72 Hz, 1H, OH-3´´), 5.33 (d, *J=* 4.88 Hz, 1H, OH-2´´), 5.50 (dd, *J=* 12.6, 2.48 Hz, 1H, H-2), 6.13 (d, *J=* 2.2, 1H, H-6), 6.15 (d, *J=* 1.96, 1H, H-8), 6.79 (d, *J=* 8.4 Hz, 2H, H-3´, H-5´), 7.32 (d, *J=* 8.44 Hz, 2H, H-2´, H-6´), 9.59 (s, 1H, OH-4´), 12.05 (s, 1H, OH-5).

**Fig. 18:**
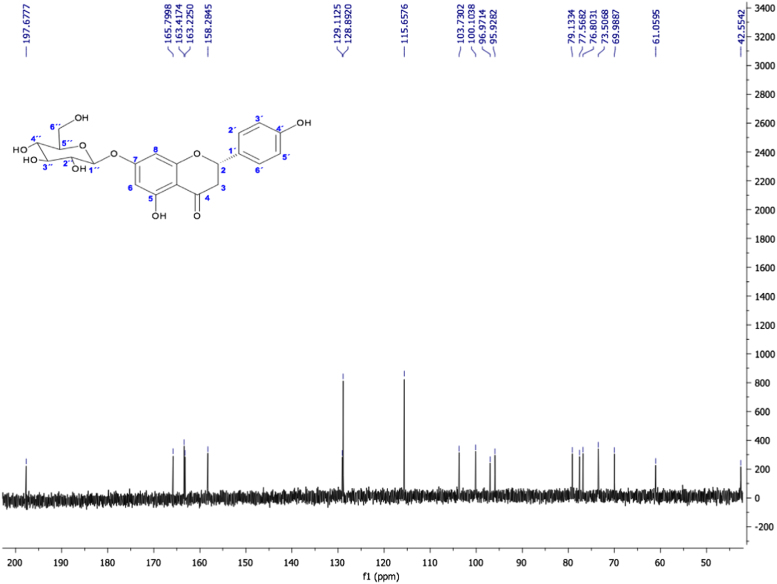
(2S) -Naringenin 7-O-β-D-glucopyranoside. NMR ^13^C (100 MHz, DMSO-d_6_) δ ppm: 42.55 (C3), 61.05 (C6´´), 69.98 (C4´´), 73.50 (C2´´), 76.80 (C3´´), 77.56 (C5´´), 79.13 (C2), 95.92 (C8), 96.97 (C6), 100.10 (C1´´), 103.73 (C10), 115.65 (C3´, C5´), 128.89 (C2´, C6´), 129.11 (C1´), 158.28 (C4´), 163.25 (C5), 163.41 (C9), 165.79 (C7), 197. 67 (C4).

**Fig. 19:**
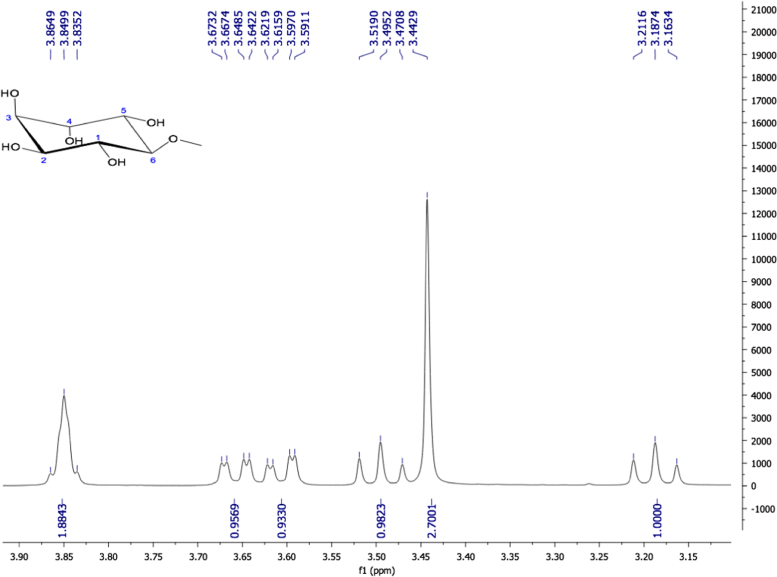
Pinitol, NMR ^1^H (400 MHz, D_2_O) δ (ppm): 3.18 (t, *J=*9.64 Hz, 1H, H-6), 3.44 (s, 3H, OCH_3_), 3.49 (t, *J=*9.64 Hz, 1H, H-1), 3.55 (dd, *J=*9.94, 2.38 Hz, 1H, H-2), 3.65 (dd, *J=*9.98, 2.42 Hz, 1H, H-5), 3.84 (m, 2H, H-3, H-4).

**Fig. 20:**
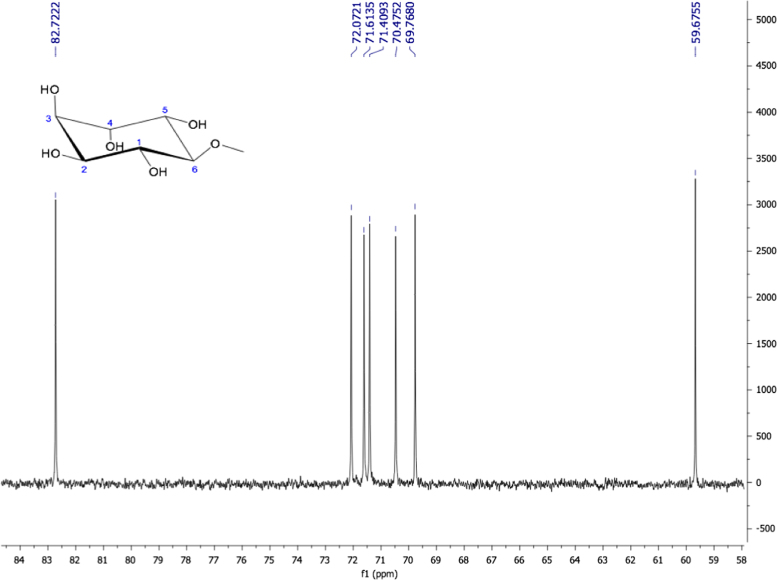
Pinitol. NMR ^13^C (100 MHz, D_2_O) δ (ppm): 59.67 (OCH_3_), 69.76 (C5), 70.47 (C2), 71.40 (C3), 71.61 (C4), 72.07 (C1), 82.72 (C6).

**Fig. 21:**
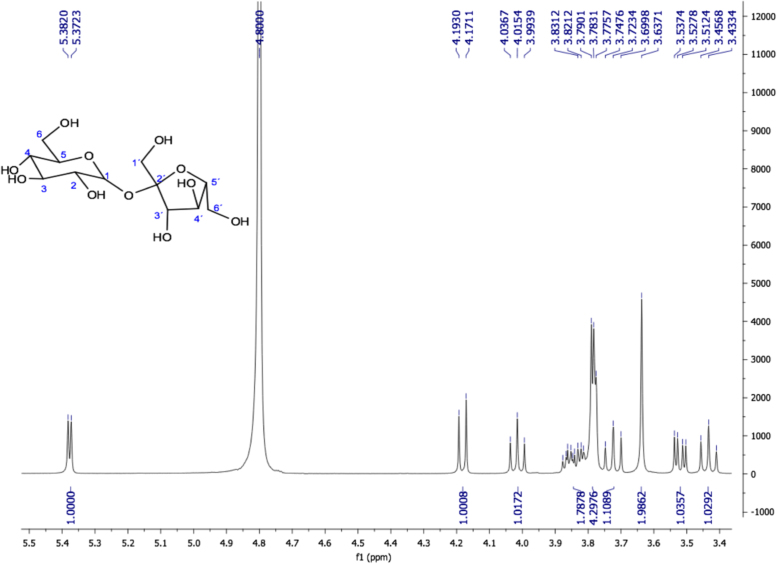
Sucrose, NMR ^1^H (400 MHz, D_2_O) δ (ppm): 3.43 (t, *J=*9.42 Hz, 1H, H-4), 3.52 (dd, *J=*10, 3.84 Hz, 1H, H-2), 3.63 (s, 2H, H-1´), 3.72 (t, *J=*9.56 Hz, 1H, H-3), 3.78 (d, *J=*2.96 Hz, 2H, H-6), 3.79 (d, *J=*2.8 Hz, 2H, H-6´), 3.83 (m, 1H, H-5), 3.86 (m, 1H, H-5´), 4.01 (t, *J=* 8.56 Hz, 1H, H-4´), 4.18 (d, *J=*8.76 Hz, 1H, H-3´), 5.38 (d, *J=*3.88 Hz, 1H, H-1).

**Fig. 22:**
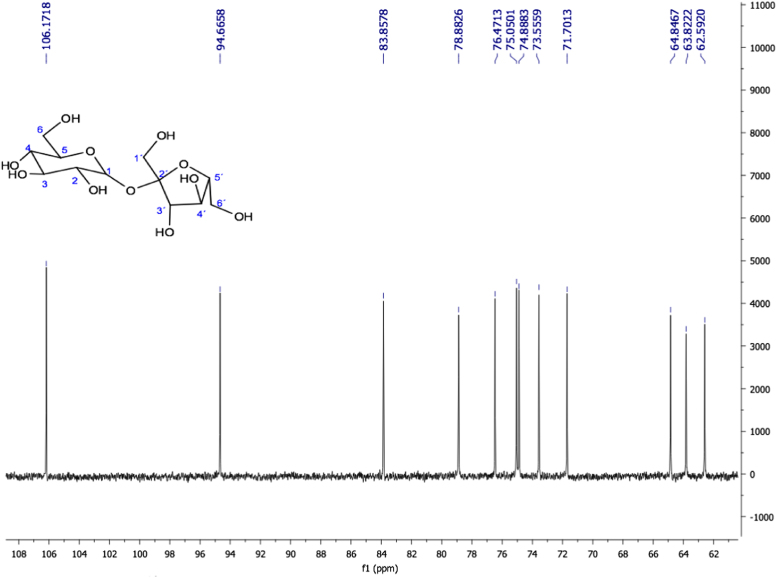
Sucrose. NMR ^13^C (100 MHz, D_2_O) δ (ppm): 62.59 (C6), 63.82 (C1´), 64.84 (C6´), 71.70 (C4), 73.55 (C2), 74.88 (C5), 75.05 (C3), 76.47 (C4´), 78.88 (C3´), 83.85 (C5´), 94.66 (C1), 106.17 (C2´).

## 2 Experimental design, materials and methods

One-dimensional nuclear magnetic resonance (NMR) spectra were obtained using the Bruker AVANCE III HD 400 MHz equipment. Deuterated solvents (CDCl_3_, DMSO-d_6_, acetone-d_6_ and D_2_O) were used based on the dissolution needs of the compounds to be studied and tetramethylsilane (TMS) as internal standard.

5–10 mg of each compound analyzed was weighed in analytical balance and 0.5 mL of deuterated solvent was added to sample until complete solubility. Then solution was placed in a clean and dry resonance tube.

To obtain the spectroscopic data of hydrogen nucleus (^1^H), a 400 MHz equipment frequency was used, while for the carbon nucleus (^13^C) a frequency of 100 MHz was used.
