# Supplementary material for: Nuclear magnetic resonance spectroscopy data of isolated compounds from Acacia farnesiana (L) Willd fruits and two esterified derivatives
Source: Data Brief. 2018 Dec 7;22:255–68. doi: 10.1016/j.dib.2018.12.008 (PMC6305907; doi:10.1016/j.dib.2018.12.008)
Supplement: Supplementary file 1 — Supplementary material. [file mmc1.pdf]

CONFLICT OF INTEREST STATEMENT

December 3, 2018

Dear DIB Managing Editor,

With regards to our DIB-D-18-02884 manuscript entitled "Nuclear Magnetic Resonance Spectroscopy Data of isolated compounds from *Acacia farnesiana* (L) Willd fruits and two esterified derivatives" submitted to Data in Brief, we have provided a statement below confirming that there are no conflicts of interest.

There are no known conflicts of interest associated with this publication and there has been no significant financial support for this work that could have influenced its outcome. The manuscript has been read and approved by all authors and the regulations of our institution concerning intellectual property have been followed.

Sincerely,

Prof. María del Rayo Camacho Corona

División de Estudios de Posgrado, Facultad de Ciencias Químicas Universidad Autónoma de Nuevo León. Guerreo y Progreso S/N. Col. Treviño, Monterrey, Nuevo León, México. C.P. 64570. Phone: +528183294000 ext 3414; FAX:+528183529025; e-mail: maria.camachocn@uanl.edu.mx
